# (2*S*,3*R*,4*R*,5*R*)-3,4-Dihydr­oxy-5-(hydroxy­meth­yl)pyrrolidine-2-carboxylic acid [(2*S*,3*R*,4*R*,5*R*)-3,4-dihydr­oxy-5-(hydroxy­meth­yl)proline]

**DOI:** 10.1107/S1600536809035636

**Published:** 2009-09-09

**Authors:** Daniel Best, Sarah F. Jenkinson, Amber L. Thompson, David J. Watkin, Francis X. Wilson, Robert J. Nash, George W. J. Fleet

**Affiliations:** a Department of Organic Chemistry, Chemistry Research Laboratory, Department of Chemistry, University of Oxford, Oxford OX1 3TA, England; b Department of Chemical Crystallography, Chemistry Research Laboratory, Department of Chemistry, University of Oxford, Oxford OX1 3TA, England; c Summit PLC, 91 Milton Park, Abingdon, Oxon OX14 4RY, England; d Phytoquest Limited, IBERS, Aberystwyth University, Plas Gogerddan, Aberystwyth SY23 3EB, Ceredigion, Wales

## Abstract

The crystal structure of the title compound, C_6_H_11_NO_5_, establishes the relative configuration at the four stereogenic centres; the absolute configuration is determined by the use of d-glucuronolactone as the starting material for the synthesis. Mol­ecules are linked by inter­molecular O—H⋯O and N—H⋯O hydrogen bonds into a three-dimensional network, with each mol­ecule acting as a donor and acceptor for five hydrogen bonds.

## Related literature

For related literature on imino­sugars, see: Asano *et al.* (2000 ▶); Watson *et al.* (2001 ▶). For related literature on pipecolic acids, see: Fleet *et al.* (1987 ▶); Booth *et al.* (2007 ▶); Bashyal, Chow, Fellows & Fleet (1987 ▶); Manning *et al.* (1985 ▶); di Bello *et al.* (1984 ▶); Yoshimura *et al.* (2008 ▶). For related literature on bulgecinine, see: Toumi *et al.* (2008 ▶); Bashyal *et al.* (1986 ▶); Bashyal, Chow & Fleet (1987 ▶); Shinagawa *et al.* (1984 ▶, 1985 ▶). For related literature on alexines, see: Pereira *et al.* (1991 ▶); Donohoe *et al.* (2008 ▶); Kato *et al.* (2003 ▶); Wormald *et al.* (1998 ▶). For absolute configuration, see: Flack (1983 ▶); Flack & Bernardinelli (2000 ▶); Flack & Shmueli (2007 ▶); Hooft *et al.* (2008 ▶); Thompson *et al.* (2008 ▶); Watkin (1994 ▶). For the weighting scheme, see: Prince (1982 ▶); Thompson & Watkin (2009 ▶).
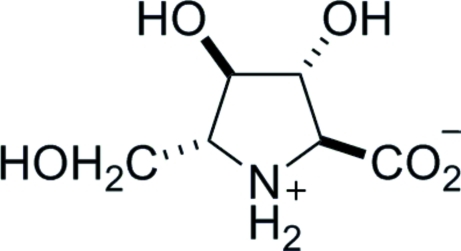


## Experimental

### 

#### Crystal data

C_6_H_11_NO_5_
*M* *_r_* = 177.16
Triclinic, 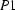

*a* = 5.4160 (2) Å
*b* = 5.8236 (3) Å
*c* = 6.6006 (3) Å
α = 102.836 (2)°
β = 104.776 (2)°
γ = 102.8244 (19)°
*V* = 187.50 (2) Å^3^
*Z* = 1
Mo *K*α radiation
μ = 0.14 mm^−1^
*T* = 150 K
0.25 × 0.17 × 0.06 mm

#### Data collection

Area diffractometer
Absorption correction: multi-scan (*DENZO*/*SCALEPACK*; Otwinowski & Minor, 1997 ▶) *T*_min_ = 0.94, *T*_max_ = 0.99
2314 measured reflections
834 independent reflections
814 reflections with *I* > 2σ(*I*)
*R*_int_ = 0.025

#### Refinement

*R*[*F* ^2^ > 2σ(*F* ^2^)] = 0.027
*wR*(*F* ^2^) = 0.066
*S* = 1.00
834 reflections
109 parameters
3 restraints
H-atom parameters constrained
Δρ_max_ = 0.24 e Å^−3^
Δρ_min_ = −0.17 e Å^−3^

### 

Data collection: *COLLECT* (Nonius, 2001 ▶); cell refinement: *DENZO*/*SCALEPACK* (Otwinowski & Minor, 1997 ▶); data reduction: *DENZO*/*SCALEPACK*; program(s) used to solve structure: *SIR92* (Altomare *et al.*, 1994 ▶); program(s) used to refine structure: *CRYSTALS* (Betteridge *et al.*, 2003 ▶); molecular graphics: *CAMERON* (Watkin *et al.*, 1996 ▶); software used to prepare material for publication: *CRYSTALS*.

## Supplementary Material

Crystal structure: contains datablocks I, global. DOI: 10.1107/S1600536809035636/lh2896sup1.cif

Structure factors: contains datablocks I. DOI: 10.1107/S1600536809035636/lh2896Isup2.hkl

Additional supplementary materials:  crystallographic information; 3D view; checkCIF report

## Figures and Tables

**Table 1:** Hydrogen-bond geometry (Å, °)

*D*—H⋯*A*	*D*—H	H⋯*A*	*D*⋯*A*	*D*—H⋯*A*
O8—H81⋯O1^i^	0.83	1.85	2.679 (3)	175
N5—H51⋯O11^ii^	0.88	2.03	2.873 (3)	160
N5—H52⋯O3^iii^	0.89	1.93	2.814 (3)	173
O11—H111⋯O1^iii^	0.82	1.89	2.696 (3)	170
O12—H121⋯O8^iv^	0.82	1.91	2.668 (3)	154

Symmetry codes: (i) 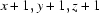; (ii) 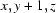; (iii) 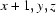; (iv) 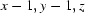.

**Fig. 1.**
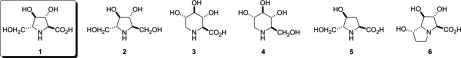
Synthetic scheme.

**Fig. 2.**
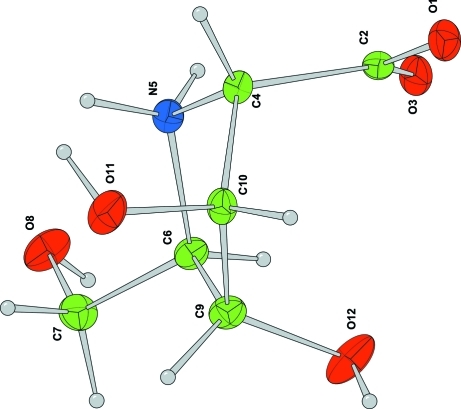
The title compound with displacement ellipsoids drawn at the 50% probability level. H atoms are shown as spheres of arbitary radius.

**Fig. 3.**
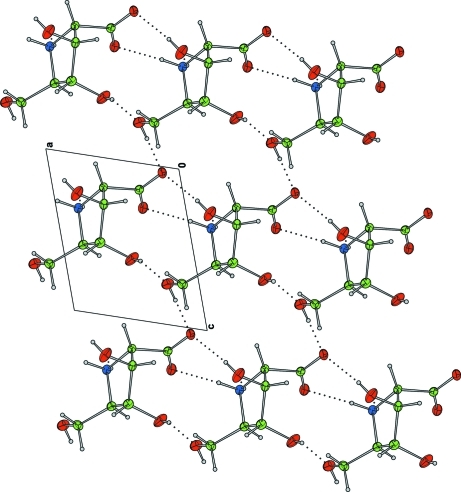
Packing diagram for the title compound projected along the b-axis.

**Fig. 4.**
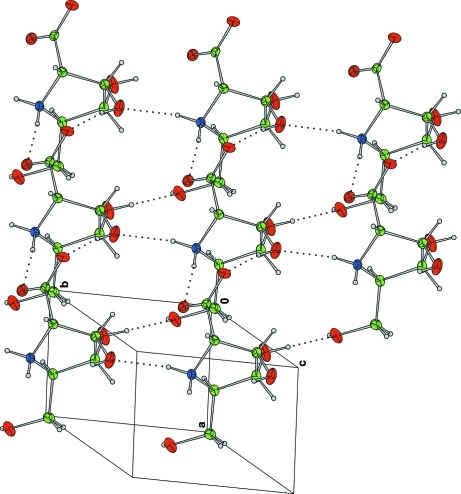
Packing diagram for the title compound. The compound exists as an extensively hydrogen bonded nextwork.

## supplementary crystallographic information

### Comment

This paper firmly establishes the structure of the trihydroxyproline 1 (Fig. 1), the amino acid corresponding to DMDP 2. There are over 100 iminosugars that have been isolated as natural products [such as DMDP 2 and DNJ 4] that are the equivalent of carbohydrates with the ring oxygen replaced by nitrogen (Asano *et al.*, 2000; Watson *et al.*, 2001). In contrast, the pipecolic acid BR1 3 [related to DNJ 4 in the same way as 1 to 2] (Fleet *et al.*,1987; Booth *et al.*, 2007; Bashyal, Chow, Fellows & Fleet, 1987) is among the rare examples of naturally occurring amino acid sugar analogues. BR1 3 was isolated from the seeds of Baphia racemosa (Manning *et al.*, 1985) and is an inhibitor of glucuronidase and iduronidase (di Bello *et al.*, 1984; Yoshimura *et al.*, 2008). Bulgecinine 5 (Toumi *et al.*, 2008; Bashyal *et al.*, 1986; Bashyal, Chow & Fleet, 1987), a deoxy analogue of 1, is a constituent of the bulgecin glycopeptide antibiotics (Shinagawa *et al.*, 1984; Shinagawa *et al.*, 1985). 7a-Epialexaflorine 6, isolated from the leaves of Alexa grandiflora (Pereira *et al.*, 1991), is the only example of an amino acid analogue of the alexines (Donohoe *et al.*, 2008; Kato *et al.*, 2003; Wormald *et al.*, 1998).

The title compound (Fig. 2) was seen to adopt an envelope conformation with C4 out of the plane. The absolute configuration was determined by the use of D-glucuronolactone as the starting material for the synthesis. The molecule exists as an extensively hydrogen bonded nextwork with each molecule acting as a donor and acceptor for 5 hydrogen bonds (Fig. 3, Fig. 4). Only classical hydrogen bonding has been considered.

### Experimental

The title compound was recrystallized from a mixture of hot ethanol and water: m.p. 449 K - decomposed; [α]_D_^25^ +14.7 (*c*, 1.13 in H_2_O).

### Refinement

Initial refinement of the Flack *x* parameter gave a value of -0.5 (10), suggesting that the absolute configuration could not be determined (Flack, 1983; Flack & Bernardinelli, 2000). Analysis of the Bijvoet differences using *CRYSTALS* gave the Hooft *y* parameter as -0.2 (7), and the probability the configuration is correct assuming the material is enantiopure was determioned to be 78.7% (Hooft *et al.*, 2008; Thompson *et al.* 2008; Thompson & Watkin 2009). In the absence of significant anomalous scattering (FRIEDIF = 6.71; Flack & Shmueli, 2007), Friedel pairs were merged for the final refinement.

The H atoms were all located in a difference map, but those attached to carbon atoms were repositioned geometrically. The H atoms were initially refined with soft restraints on the bond lengths and angles to regularize their geometry (C—H in the range 0.93–0.98, N—H in the range 0.86–0.89 N—H to 0.86 O—H = 0.82 Å) and *U*_iso_(H) (in the range 1.2–1.5 times *U*_eq_ of the parent atom), after which the positions were refined with riding constraints.

### Crystal data

**Table:**

C_6_H_11_NO_5_	*Z* = 1
*M**_r_* = 177.16	*F*(000) = 94
Triclinic, *P*1	*D*_x_ = 1.569 Mg m^−^^3^
Hall symbol: P 1	Melting point: not measured K
*a* = 5.4160 (2) Å	Mo *K*α radiation, λ = 0.71073 Å
*b* = 5.8236 (3) Å	Cell parameters from 696 reflections
*c* = 6.6006 (3) Å	θ = 5–27°
α = 102.836 (2)°	µ = 0.14 mm^−^^1^
β = 104.776 (2)°	*T* = 150 K
γ = 102.8244 (19)°	Plate, clear_pale_colourless
*V* = 187.50 (2) Å^3^	0.25 × 0.17 × 0.06 mm

### Data collection

**Table:**

Area diffractometer	814 reflections with *I* > 2σ(*I*)
graphite	*R*_int_ = 0.025
ω scans	θ_max_ = 27.5°, θ_min_ = 5.6°
Absorption correction: multi-scan (*DENZO*/*SCALEPACK*; Otwinowski & Minor, 1997)	*h* = −7→7
*T*_min_ = 0.94, *T*_max_ = 0.99	*k* = −6→7
2314 measured reflections	*l* = −8→7
834 independent reflections	

### Refinement

**Table:**

Refinement on *F*^2^	Primary atom site location: structure-invariant direct methods
Least-squares matrix: full	Hydrogen site location: inferred from neighbouring sites
*R*[*F*^2^ > 2σ(*F*^2^)] = 0.027	H-atom parameters constrained
*wR*(*F*^2^) = 0.066	Method, part 1, Chebychev polynomial, (Watkin, 1994, *P*rince, 1982) [*w*eight] = 1.0/[A_0_*T_0_(x) + A_1_*T_1_(x) ··· + A_n-1_]*T_n-1_(x)] where A_i_ are the Chebychev coefficients listed belo*w* and x = *F* /*F*max Method = Robust Weighting (*P*rince, 1982) W = [*w*eight] * [1-(delta*F*/6*sigma*F*)^2^]^2^ A_i_ are: 22.5 35.8 21.7 10.1 2.91
*S* = 1.00	(Δ/σ)_max_ = 0.0001
834 reflections	Δρ_max_ = 0.24 e Å^−^^3^
109 parameters	Δρ_min_ = −0.17 e Å^−^^3^
3 restraints	

### Fractional atomic coordinates and isotropic or equivalent isotropic displacement parameters (Å^2^)

**Table:**

	*x*	*y*	*z*	*U*_iso_*/*U*_eq_	
O1	0.1323 (3)	0.4021 (3)	0.0397 (3)	0.0170	
C2	0.3330 (4)	0.5769 (4)	0.1694 (3)	0.0124	
O3	0.3285 (3)	0.7782 (3)	0.2816 (3)	0.0161	
C4	0.6067 (4)	0.5299 (3)	0.1892 (3)	0.0118	
N5	0.8297 (3)	0.7483 (3)	0.3462 (3)	0.0120	
C6	0.8425 (4)	0.7309 (4)	0.5744 (3)	0.0130	
C7	1.1277 (4)	0.8313 (4)	0.7284 (3)	0.0167	
O8	1.2417 (3)	1.0809 (3)	0.7415 (3)	0.0209	
C9	0.7092 (4)	0.4584 (4)	0.5443 (3)	0.0150	
C10	0.6269 (4)	0.3243 (4)	0.2968 (3)	0.0135	
O11	0.8192 (3)	0.2063 (3)	0.2544 (3)	0.0229	
O12	0.4844 (4)	0.4511 (3)	0.6175 (3)	0.0252	
H41	0.6365	0.4960	0.0477	0.0144*	
H61	0.7333	0.8262	0.6278	0.0169*	
H72	1.1284	0.8203	0.8747	0.0191*	
H71	1.2343	0.7354	0.6770	0.0194*	
H91	0.8360	0.3904	0.6257	0.0183*	
H101	0.4566	0.2013	0.2549	0.0154*	
H81	1.2048	1.1744	0.8368	0.0313*	
H51	0.7954	0.8869	0.3332	0.0177*	
H52	0.9817	0.7443	0.3204	0.0178*	
H111	0.8993	0.2734	0.1839	0.0347*	
H121	0.4206	0.3112	0.6224	0.0383*	

### Atomic displacement parameters (Å^2^)

**Table:**

	*U*^11^	*U*^22^	*U*^33^	*U*^12^	*U*^13^	*U*^23^
O1	0.0115 (7)	0.0187 (7)	0.0178 (7)	0.0024 (5)	0.0034 (5)	0.0030 (6)
C2	0.0128 (9)	0.0137 (9)	0.0134 (9)	0.0052 (7)	0.0051 (7)	0.0074 (7)
O3	0.0153 (7)	0.0148 (7)	0.0210 (7)	0.0075 (6)	0.0074 (5)	0.0057 (6)
C4	0.0113 (9)	0.0115 (8)	0.0130 (9)	0.0032 (7)	0.0050 (7)	0.0032 (7)
N5	0.0123 (8)	0.0098 (7)	0.0150 (8)	0.0039 (6)	0.0052 (6)	0.0042 (6)
C6	0.0142 (9)	0.0115 (8)	0.0136 (9)	0.0030 (7)	0.0053 (7)	0.0041 (7)
C7	0.0154 (9)	0.0161 (9)	0.0152 (9)	0.0013 (8)	0.0023 (7)	0.0042 (8)
O8	0.0246 (8)	0.0151 (8)	0.0190 (7)	−0.0019 (6)	0.0100 (6)	0.0020 (6)
C9	0.0173 (10)	0.0130 (9)	0.0165 (10)	0.0042 (7)	0.0066 (8)	0.0067 (7)
C10	0.0121 (9)	0.0124 (9)	0.0176 (9)	0.0049 (7)	0.0058 (7)	0.0050 (7)
O11	0.0280 (8)	0.0223 (8)	0.0356 (9)	0.0180 (7)	0.0218 (7)	0.0179 (7)
O12	0.0325 (9)	0.0164 (7)	0.0304 (9)	0.0018 (7)	0.0222 (8)	0.0056 (7)

### Geometric parameters (Å, °)

**Table:**

O1—C2	1.264 (3)	C7—O8	1.421 (2)
C2—O3	1.249 (2)	C7—H72	0.981
C2—C4	1.545 (2)	C7—H71	0.959
C4—N5	1.498 (2)	O8—H81	0.832
C4—C10	1.532 (3)	C9—C10	1.545 (3)
C4—H41	0.972	C9—O12	1.415 (2)
N5—C6	1.517 (2)	C9—H91	0.972
N5—H51	0.885	C10—O11	1.420 (2)
N5—H52	0.886	C10—H101	0.963
C6—C7	1.515 (3)	O11—H111	0.816
C6—C9	1.535 (3)	O12—H121	0.823
C6—H61	0.973		
			
O1—C2—O3	126.24 (18)	C6—C7—O8	111.22 (16)
O1—C2—C4	115.43 (17)	C6—C7—H72	108.8
O3—C2—C4	118.32 (17)	O8—C7—H72	109.4
C2—C4—N5	110.92 (15)	C6—C7—H71	109.8
C2—C4—C10	109.46 (14)	O8—C7—H71	108.5
N5—C4—C10	103.55 (15)	H72—C7—H71	109.1
C2—C4—H41	111.2	C7—O8—H81	110.0
N5—C4—H41	108.5	C6—C9—C10	106.41 (15)
C10—C4—H41	113.0	C6—C9—O12	106.33 (16)
C4—N5—C6	106.33 (14)	C10—C9—O12	111.81 (16)
C4—N5—H51	110.5	C6—C9—H91	110.0
C6—N5—H51	109.1	C10—C9—H91	109.3
C4—N5—H52	109.8	O12—C9—H91	112.7
C6—N5—H52	111.2	C9—C10—C4	103.65 (15)
H51—N5—H52	109.8	C9—C10—O11	109.89 (16)
N5—C6—C7	110.92 (15)	C4—C10—O11	113.98 (15)
N5—C6—C9	105.61 (15)	C9—C10—H101	108.3
C7—C6—C9	114.30 (16)	C4—C10—H101	111.8
N5—C6—H61	108.0	O11—C10—H101	109.0
C7—C6—H61	109.9	C10—O11—H111	110.0
C9—C6—H61	107.9	C9—O12—H121	109.5

### Hydrogen-bond geometry (Å, °)

**Table:**

*D*—H···*A*	*D*—H	H···*A*	*D*···*A*	*D*—H···*A*
C7—H71···O12^i^	0.96	2.39	3.328 (3)	166
C10—H101···O3^ii^	0.96	2.47	3.199 (3)	133
O8—H81···O1^iii^	0.83	1.85	2.679 (3)	175
N5—H51···O11^iv^	0.88	2.03	2.873 (3)	160
N5—H52···O3^i^	0.89	1.93	2.814 (3)	173
O11—H111···O1^i^	0.82	1.89	2.696 (3)	170
O12—H121···O8^v^	0.82	1.91	2.668 (3)	154

Symmetry codes: (i) *x*+1, *y*, *z*; (ii) *x*, *y*−1, *z*; (iii) *x*+1, *y*+1, *z*+1; (iv) *x*, *y*+1, *z*; (v) *x*−1, *y*−1, *z*.
